# Health-Promoting Effects of *Thymus* Phenolic-Rich Extracts: Antioxidant, Anti-inflammatory and Antitumoral Properties

**DOI:** 10.3390/antiox9090814

**Published:** 2020-09-01

**Authors:** Andrea F. Afonso, Olívia R. Pereira, Susana M. Cardoso

**Affiliations:** 1 LAQV-REQUIMTE, Department of Chemistry, University of Aveiro, 3810-193 Aveiro, Portugal; andrea@ipb.pt; 2 Public Health Laboratory of Bragança, Local Health Unit, Rua Eng. Adelino Amaro da Costa, 5300-146 Bragança, Portugal; 3 Centro de Investigação de Montanha (CIMO), Instituto Politécnico de Bragança, Campus de Santa Apolónia, 5300-253 Bragança, Portugal; oliviapereira@ipb.pt

**Keywords:** *Thymus*, antioxidant, anti-inflammatory, antitumoral, phenolic compounds, rosmarinic acid

## Abstract

*Thymus* genus comprises numerous species that are particularly abundant in the West Mediterranean region. A growing body of evidence suggests that many of these species are a rich source of bioactive compounds, including phenolic compounds such as rosmarinic acid, salvianolic acids and luteolin glycosides, able to render them potential applications in a range of industrial fields. This review collects the most relevant studies focused on the antioxidant, anti-inflammatory and anti-cancer of phenolic-rich extracts from *Thymus* plants, highlighting correlations made by the authors with respect to the main phenolic players in such activities.

## 1. Introduction

*Thymus* is a Latin name of the plant genus, which is considered to derive from the Greek word *thyo* (perfume) by some authors, while others consider the Greek word *thymos* (courage, strength) [1]. *Thymus* genus belongs to Lamiaceae family and includes about 350 aromatic species that are distributed around the world, with particularly abundancy in the West Mediterranean region [2,3]. These species are perennial and are characterized as being herbaceous subshrubs or shrubs with 10 to 30 cm tall, containing small and simple leaves, ramified and prostrated branches and big clusters of pink, white, cream or violet flowers [2].

*Thymus* plants are claimed to be endowed of distinct health beneficial properties, including antimicrobial, antioxidant, anti-inflammatory, cardioprotective, neuroprotective, anticarcinogenic and hypoglycemic activities [4,5,6,7]. Although these benefits have been mostly associated with essential oils [8,9,10,11], nowadays, *Thymus* phenolic-rich extracts usually obtained with polar solvents, are an attractive target for the screening of bioactive compounds for possible industrial applications in distinct fields, including food, cosmetics or pharmaceutical industries [6,12]. Among them, counteraction of oxidative stress events is a central issue underlying the valorization of these plants.

Oxidative stress is generated when the balance between reactive species formation and their elimination is disrupted. Under this biological condition, there is an overproduction of reactive oxygen species (ROS) such as superoxide (O_2_^-^), hydroxyl radical (^●^OH) and hydrogen peroxide (H_2_O_2_), as well as of reactive nitrogen species (RNS) like nitric oxide (NO) and peroxynitrite (ONOO^¯^), overall leading to several mitochondrial and cellular damages. These events are closely associated to aging processes [13,14] and to the physiopathology of several oxidative-related diseases [15,16,17]. To minimize the oxidative damage, enzymatic and non-enzymatic antioxidants are involved. The first include superoxide dismutase (SOD), which catalyzes the conversion of O_2_^-^ to H_2_O_2_, which in its turn, can be neutralized by catalase or glutathione peroxidase (GPx), while non-enzymatic antioxidants enclose, among others, vitamins A, C and E, the glutathione reduced form (GSH) and β-carotene [16,17,18,19]. Oxidative stress also plays a critical role in the triggering and progression of inflammation, through the overproduction of ROS and RNS. In this process, the enzyme NO synthase (iNOS) is responsible for the production of NO, which is a key free radical in inflammatory events. Globally, these oxidative agents result from activation of immunological cells that can also generate proinflammatory agents [18,20], namely chemokines, interleukins (IL) and interferons (INF), which are majorly regulated by the transcriptional factor kappa B (NF-κB). The activation of adherent molecules by chemokines and complement split products is also triggered, enabling leukocytes migration into tissue spaces and the release of pro-inflammatory cytokines (e.g., IL-1β, IL-6, IL-12 and tumor necrosis factor (TNF-α)), that in turn stimulate and amplify inflammation [18,20,21]. Moreover, there is the activation of arachidonic acid, whose metabolism generates prostaglandins and tromboxanes by the cyclooxygenases pathway and leukotrienes by lipoxygenase pathway, stimulated by the enzymes cyclooxygenases (constitutive COX-1 and inducible COX-2) and by 5-lipoxygenase (5-LOX), respectively [22,23,24].

Notably, oxidative stress and chronic inflammation are also known to contribute to malignant transformation of cells associated with tumor cell proliferation and tumor progression, as well as angiogenesis and tissue remodeling, induction of neoplastic transformation and metastasis [16,25,26]. The cancerous state develops when the normal tissue homeostasis is broken, resulting in an imbalance between cellular proliferation and programmed cell death (apoptosis). It is known that apoptosis can be induced by activation of the caspase cascade through death receptors pathway or the mitochondrial death signaling, whose members are either proapoptotic (Bax, Bad) or antiapoptotic (Bcl-2, Bcl-XL) [27,28,29]. Therefore, the induction of programed cell death in cancer cells is one of the strategies used in the development of anti-cancer drugs [24,30,31,32] and, in particular, plants used in traditional forms have been the focus of attention as plant-based foods, whose regular consumption may be correlated with a lower incidence of cancer [33,34,35,36].

In recent years, numerous studies have reported the bioactivities of different species belonging to the *Thymus* genus. This review represents the first collection of data regarding the antioxidant, anti-inflammatory and antitumoral properties of phenolic-rich extracts from *Thymus*, highlighting its health-benefits and relevance as potential agents with pharmacological activity. 

## 2. Antioxidant Activity 

The antioxidant activity of *Thymus* species has been closely associated with their phenolic richness and/or specific phenolic composition [37] and hence, many authors have previously screened the antioxidant potential of polar extracts. The majority of such studies used one or several chemical methods, including the (i) scavenging of stable free radicals such as DPPH^●^ (2.2-diphenyl-1-picrylhydrazyl), ABTS^●+^ (2.2′-azino-bis-3-ethylbenzthiazoline-6-sulphonic acid), ^●^OH, O_2_^-^ and NO; (ii) the reduction of metal ions from the higher to the lower oxidation state by reducing power method (RP) and ferric reducing antioxidant power assay (FRAP); (iii) the oxygen radical absorbance capacity (ORAC), which measures the radical chain breaking ability of antioxidants by the inhibition of peroxyl radical (RO_2_^•^)–induced oxidation and trolox equivalence antioxidant capacity (TEAC); (iv) the inhibition of lipid peroxidation by ß-carotene bleaching test; and (v) the inhibition of lipid peroxidation by decreasing the thiobarbituric acid reactive substances (TBARS). The extensive use of such assays is mainly attributed to their simplicity and quickness of execution [38,39]. In general, these methods are accepted as a first approach to screen the antioxidant potential of the samples when used combined (because there is no universal method for antioxidant ability screening) and compared to positive controls [4,40,41,42,43,44]. Studies complying such criteria are summarized in Table 1.

Since *Thymus vulgaris* is a widespread species, its polar extracts have been explored for a range of biological effects, including antioxidant ability. Among the reported data for this species, water, methanolic and hydroalcoholic extracts of this plant origin were reported to have DPPH^●^ EC_50_ values in the range of 1.8 and 44.7 μg/mL [4,57,59]. Moreover, phenolic-rich extracts from *T. vulgaris* were shown to be good ABTS^●+^ (EC_50_ = 0.7–13.8 μg/mL) [57,59] and ^●^OH (EC_50_ = 0.24 μg/mL) [57] scavenging agents (Table 1).

The promising antioxidant potential of many others *Thymus* extracts were also previously emphasized by distinct authors. In particular, a methanolic extract of *Thymus nummularius*, also known as *Thymus pseudopulegioides*, was claimed to have higher DPPH^●^ scavenging ability (EC_50_ = 5.7 μg/mL) than 3,5-di-tert-butyl-4 hydroxytoluene (BHT) (EC_50_ = 47.1 μg/mL), and to be 1.5 times more promising than this standard in ABTS^●+^ and ß-carotene bleaching inhibition methods [44]. Moreover, hydromethanolic extracts of *Thymus capitatus* [50] and *Thymus mastichina* [37], which presented rosmarinic acid as major phenolic constituent, were shown to have high antioxidant potential based in DPPH^●^ (18–149 mg TE/g DW) and FRAP assays (30–154 mg TE/g DW). 

In addition, a significant scavenging activity of ABTS^●+^ (expressed as mmol Trolox eq./g extract) was observed for hydroethanolic/aqueous decoction extracts of *Thymus pulegioides* (1.53/1.58) [61], *Thymus citriodorus* (1.52/1.21) and *T. vulgaris* (0.92/0.79) [51], *T. mastichina* (1.48/0.96) [54], and *Thymus zygis* subsp. *zygis* (1.08/0.76) [60]. These authors also noted the high inhibitory ability of some of these extracts (at 0.1 mg/mL) against hydroxyl radical in the presence of EDTA, namely the decoctions of *T. zygis* subsp. *zygis* (66%) [60] and *T. citriodorus* (37.9%) [51], as well the hydroethanolic and decoctions of *T. mastichina* (43.2 and 48.5%, respectively) [54] and *T. pulegioides* (34.8 and 34.0%, respectively) [61].

Favorable DPPH^●^ and reducing power results were also been observed by our group for decoctions of *Thymus herba-barona, Thymus pseudolanuginosus*, *Thymus caespititius*, *T. pulegioides*, *Thymus fragantissimus* and *T. zygis* [40,41], for which the EC_50_ values were 1.4–2.0 higher than those of the reference commercial compounds. Among these six extracts, those rich in flavones (*T. pulegioides* and *T. pseudolanuginosus*) tended to have superior DPPH^●^ scavenging ability and reducing Fe ability. In fact, the DPPH^●^ scavenging ability of these extracts surpassed that recently described for decoction and infusion extracts of *Thymus sipyleus* Boiss. subsp. *rosulans* (Borbas) *Jalas* (EC_50_ values of 43.5±1.02 and 87.38±1.73 μg/mL, corresponding to about 1.6–3.2-fold of the ascorbic acid). Interestingly, the authors disclosed a greater antioxidant ability of these polar solvent extracts in comparison to non-polar ones [56]. Different results were described for aqueous extracts of *Thymus atlanticus, T. zygis* and *Thymus satureioides* based on ABTS^●^^+^ scavenging assay, which corresponded to about 7.5–8.5 times those of the standard ascorbic acid [62].

In opposition to chemical assays, to our knowledge, only a few authors used cellular and/or in vivo models to screen the antioxidant potential of *Thymus* polar extracts (Table 1). Under this context, hydroethanolic extracts of *T. citriodorus* (50 μg/mL) were shown to reduce significantly the intracellular ROS formation in a potassium dichromate-stimulated HepG2 cells [52]. Moreover, Khouya et al. [63] reported the protection of *T. satureioides* against oxidative damage, by demonstrating that the addition of its crude extract or subsequent fractions (dichloromethane, ethyl acetate, methanol or aqueous) to erythrocyte suspensions resulted in an increase in haemolysis half-time (1.2 to 2.1-fold of potency compared to ascorbic acid). As regard to in vivo studies, it was only recently that Righi et al. [46] reported that the treatment of *Swiss albinos* mice with *Thymus algeriensis* hydroalcoholic extracts at 800 mg/kg bw resulted in an improvement of the antiradical ability of the plasma, as well as catalase and glutathione levels (at 400 and 800 mg/kg bw), paralleled by a decrement on the levels of malondialdehyde (200 and 400 mg/kg bw). In addition, El-Boshy et al. [58] demonstrated that supplementation of lead (Pb) intoxicated rats with ethanolic extracts from *T. vulgaris* could ameliorate antioxidant parameters and the overall toxic effects.

Notably, some authors evaluated the antiradical/antioxidant activity of *Thymus* polar extracts with regard to their phenolic content and to the presence of specific phenolic compounds [37,51], particularly with the major components (e.g., rosmarinic acid, caffeic acid, luteolin-*O*-glucoside, apigenin, naringenin) [37,43,44,50]. A recent study [51] focused the antioxidant potential of *T. vulgaris* and *T. citriodorus*, described a high correlation with their polyphenol levels with their Trolox equivalent antioxidant capacity (TEAC) (e.g., rosmarinic acid (r = 0.874), eriodictyol-glucoside (r = 0.759), luteolin-hexuronide (r = 0.734, *p* < 0.0157), and chrysoeriol-hexoside (r = 0.771)) [51]. Amongst phenolic compounds, rosmarinic acid is frequently the principal phenolic acid in the said extracts and it is believed to play a central role in their antioxidant abilities [37,44]. In fact, this caffeic acid dimer was shown to be sixteen times better than α-tocopherol [44] and twice better than trolox [4] in DPPH^●^-scavenging assay. It also exerts higher reducing power capacity (EC_50_ = 2.7 μg/mL) than trolox (EC_50_ = 6.6 μg/mL) [4] and higher ABTS^●+^ scavenging ability than BHT [44]. Despite that, according to Ertas et al. [44], this is less efficient that BHT in protecting against lipid peroxidation (EC_50_ value was 1.2-fold higher in the ß-carotene bleaching inhibition assay). Moreover, rosmarinic acid derivatives together with other non-flavonoids and flavonoid compounds (in particular luteolin, apigenin and its derivatives) may also contribute to the antioxidant capacity of *Thymus* extracts, since they are good antiradical/antioxidants as evaluated in chemical and cellular models. Namely, luteolin and apigenin (50 μg/mL) were demonstrated to drastically decrease the levels of intracellular ROS under oxidative-stress conditions on a hepatic cellular model [52]. Moreover, luteolin was highlighted by its high ability to prevent lipid peroxidation events when compared to rosmarinic acid [4]. 

## 3. Anti-Inflammatory Activity

A growing body of experimental evidence suggests that plant extracts or their bioactive compounds (e.g., phenolic compounds) have potential therapeutic application in the treatment of several inflammatory diseases, through distinct mechanisms, including the inhibition of pro-inflammatory cytokines, intercellular adhesion molecules and enzymes (e.g., iNOS, COX-2, 5-LOX), as well as, transcription factors, such as NF-κB [20,64,65,66,67], prevention of protein denaturation and stabilization of the potential of erythrocyte membrane [62] or control of the levels of radicals like NO [4,40]. Distinct authors evaluated the anti-inflammatory potential of *Thymus* polar extracts towards such events (Table 2). 

The capacity to inhibit key enzymes in inflammation was demonstrated for extracts of different *Thymus* species. Among them, diethyl ether, ethyl acetate and *n*-butanol extracts of *Thymus camphoratus, Thymus carnosus* and *T. mastichina* [49] and methanolic extracts of *T. algeriensis* and *Thymus fontanesii* [68] were demonstrated to possess high inhibitory capacity against 5-LOX, with the last two showing similar potency to that of the positive control zileuton, and about one third of the potency of celecoxibe, with regard to the ability to inhibit COX-2 [68]. Moreover, the anti-inflammatory activity of aqueous extracts of *T. atlanticus, T. zygis* and *T. satureioides* was evidenced because of their potency to prevent protein denaturation and cellular lysis in a dose-dependent manner [62], as shown in Table 2. Among them, the authors underlined the erythrocyte-protective effect of *T. atlanticus*, which was superior to that of indomethacin (IC_50_ of 93.28 *vs* 97.83 μg/mL). Albeit the authors did not analyze the phenolic composition of the samples, they suggested that the anti-inflammatory effects were due to its high quantity of polyphenols found in these *Thymus* species [62]. 

The ability of natural compounds in scavenging the radical NO is frequently estimated through chemical assays and this is also the case of *Thymus* polar extracts. Among the distinct studies, one must emphasize the potential of ethanolic extracts from six *Thymus* species that showed EC_50_ NO scavenging results 1-3 times higher than the tested standard compound (Trolox), with the best results being obtained for the *T. pulegioides* and *Thymus longicaulis* species (EC_50_ of 69.8 and 71.6 μg/mL, respectively) [4]. In addition, *T. herba-barona, T. pseudolanuginosus* and *T. caespititius* decoctions, which were rich in caffeic acid derivatives and flavones, were all reported as good NO scavenging agents, from which that from *T. caespititius* origin had similar potency as ascorbic acid [40].

Studies on cellular models also support the hypothesis that *Thymus* phenolic-rich extracts possess anti-inflammatory activity. Among them, the work of Ustuner and colleagues [56] underlined the anti-inflammatory potential of *T. sipyleus* Boiss. subsp. *rosulans* (Borbas) *Jalas* aqueous extract through reducing the overproduction of NO and the expression of TNF-α, as attested in lipopolysaccharide (LPS)-activated RAW 264.7 macrophages. Using the same model, Silva et al. [60] demonstrated that 50 µg/mL of hydroethanolic and aqueous extracts of *T.* *zygis* decreased the NO levels by ~89% and 48%, respectively, while the aqueous extracts of *T. carnosus Boiss.* lowered the levels of this radical in a dose-dependent manner, reaching 90% inhibition for 200 μg/mL [69]. Besides, an hydroalcoholic extract of *T. longicaulis* (rich in rosmarinic acid) was shown to effectively inhibit COX-2 gene expression in THP-1 macrophages [42] and the aqueous extract of *T. vulgaris* were attested to decrease the release of the cytokine IL-8 for both H_2_O_2_ or TNFα-stimulated peripheral blood lymphocytes (PBL) [71]. In addition, a commercial propylene glycol extract of *T. vulgaris* was reported to inhibit the production of cytokines IL-1β and TNF-α in LPS-activated RAW 264.7 macrophages after exposure to different concentrations of propylene glycol extracts [72].

Notably, some authors emphasized a straight correlation between the high levels of phenolic acids and/or flavonoids in the extracts with their antioxidant/anti-inflammatory activities [37,71]. Among non-flavonoids, rosmarinic acid (in general the major phenolic in *Thymus* polar extracts) and its derivatives are probably positive contributors to anti-inflammatory activities. In fact, rosmarinic acid was demonstrated to be effective (in vitro and in vivo) against the production and mRNA expression of TNF-α, IL-1β, IL-6, COX-2 and iNOS, and to downregulate proteins involved in the signaling NF-kB-pathway [73]. Moreover, ethyl rosmarinate, a structurally-modified rosmarinic acid, was claimed to be a potent inhibitor against lung inflammation, based on NO and PGE_2_ inhibition and also by decreasing of mRNA and protein expression of iNOS and COX-2, as demonstrated in LPS-induced alveolar macrophages [74]. In the same line, flavonoids have also been shown to inhibit the expression of various enzyme systems, inflammatory mediators (e.g., molecules adhesion, cytokines and chemokines) and molecules involved in signal transduction pathways implicated in the inflammatory response [7,24,75]. In particular, luteolin (a common flavone in *Thymus* plants in general), was reported to inhibit the expression of COX-2 and TNF-α secretion in IFN-γ-stimulated RAW264.7 macrophage cells, and to inhibit the phosphorylation of signal transducer and activator of transcription (STAT)-3, a crucial transcription factor of proliferation and activation of T cells [76].

Opposing to the antioxidant capacity, in vivo anti-inflammatory potential of diverse thyme polar extracts have been reported on animal models of acute inflammation (e.g., paw edema induced by carrageenan, ear edema induced by toxics as croton oil, measurement of myeloperoxidase in leucocytes and total numbers of polymorphonuclear leukocytes (LPM)) [77,78,79]. Among the reported bibliography of *Thymus* plants, the work of Khouya et al. [43] underlined that the aqueous extracts of *T. satureioides*, *T. atlanticus* and *T. zygis* (at 900 µg/ear) could suppress croton oil-induced mice ear edema, and in addition, the last two (at 50 mg/kg) also inhibited carrageenan-induced rat paw edema, with similar potency to that of the non-steroidal anti-inflammatory drug indomethacin [43]. More recently, the same authors showed that the application of the dichloromethane and ethyl acetate fractions of *T. satureioides* (1 mg/ear) caused significant decreases in ear edema volume after 4 h (1.8–2.1-fold that of positive control indomethacin (600 μg/ear)), while oral treatment with a crude extract (60 mg/kg) inhibited significantly the carrageenan-induced rat paw oedema, during the first phase (1–5 h) [63]. In the same model, a methanolic fraction of *Thymus praecox* subsp. *skorpilii* var. *skorpilii* showed moderate anti-inflammatory effect [70]. Moreover, the intraperitoneal administration of an ethanolic extract of *T. vulgaris* (50 and 100 mg/kg) in a rat model of experimental autoimmune encephalomyelitis (EAE), was able to decrease the cytokines production by splenocytes (IFN-γ, IL-6, and IL-17), while the amount of TGF-ß secretion was significantly higher [80]. The administration of hydromethanolic extracts of *T. algeriensis* and *T. fontanesii* (600 mg/kg) resulted in the reduction (62% and 52%) on carrageenan-induced leukocyte migration into the peritoneal cavity in mice model, while diclofenac (20 mg/kg) and dexamethasone (2 mg/kg) caused 39% and 30% of reduction, respectively [68]. 

It is possible that major phenolic constituents of *Thymus* plants may contribute to their anti-inflammatory effects since either rosmarinic acid, caffeic acid and flavonoides were previously reported as to effectively counteract inflammatory events in vivo models [73,76,77,81,82,83]. In particular, rosmarinic acid suppressed LPS-induced proinflammatory cytokine [73,77], inhibited the expression of COX-2 and iNOS protein in mice disease-inflammatory models [73,84], and reduced the myeloperoxidase activity compared with the LPS-treated cells, suggesting the neutrophil infiltration, which indirectly reflect the degree of inflammation [77]. Likewise, the flavone luteolin suppressed inflammatory signals including TNF-α, NF-kB, MCP-1 and IL-1β in LPS-induced cells, attenuating acute renal injury in mice [81]. In addition, the anti-inflammatory effects of luteolin on autoimmune thyroiditis mouse model was demonstrated by the reduction of infiltrating lymphocytes and downregulation of IL-6/STAT1 and STAT3 signaling pathway in thyroid glands [76]. In turn, administration of apigenin exerted a beneficial role in acute mouse pancreatitis, reducing necrosis to 100% and a significant inhibition of TNF-α expression [85].

The overall data focus on the anti-inflammatory potential of *Thymus* extracts and phenolic compounds as well [24], emphasize their potential useful in the development of alternative drugs for the treatment of a variety of diseases settled on oxidative and/or inflammatory events, including autoimmune diseases, diabetes, cancers and ageing.

## 4. Anticancer Activity

The antitumoral potency of *Thymus* extracts has been studied, underlying their potential as sources of chemotherapeutic agents, particularly in in vitro cellular models. As for drugs in general, the majority of these studies used the 3-(4,5-dimethylthiazol-2-yl)-2,5-diphenyltetrazolium bromide (MTT) assay as a first approach method to estimate cytotoxicity/growth-inhibitory activity of extracts in cancer cell lines (Table 3). This colorimetric method measures the activity of mitochondrial enzymes that reduce MTT to purple formazan, and this conversion is often used as an estimative of viable cells [30,59]. Similar principle methods are also applied by the authors, including those based on the reduction of 2,3-bis-(2-methoxy-4-nitro-5-sulfophenyl)-2H-tetrazolium-5-carboxanilide (XTT) [42,86]; or sulforhodamine B (SRB) assay, which is based on the ability of SRB to bind to protein components of cells under mild acidic conditions and that can be extracted under basic conditions, given an estimation of cell mass (extrapolated to growth activity) [87,88]. In general, literature data is presented as IC_50_ or GI_50_ values (inhibition concentration or grow inhibition, respectively), which both correspond to sample concentration able to inhibit 50% of the cell grow.

The antitumoral activity of the most widespread *Thymus* species, namely *T. vulgaris* and/or *Thymus serpyllum*, were previously screened. Commercial aqueous extracts from these two plant species were described to exhibit cytotoxic ability towards adriamycin-resistant human breast cancer cell line (MCF-7/Adr) [89]. Amongst representative compounds of the extracts (caffeic acid, rosmarinic acid, lithospermic acid, luteolin-7-*O*-glucuronide, luteolin-7-*O*-rutinoside and eriodictiol-7-*O*-rutinoside), rosmarinic acid and lithospermic acid were demonstrated to be the most active [89]. Notably, plant extracts (including *T. vulgaris* and *T. serpyllum*) proved to be more toxic against the cancer resistant cells than the analyzed phenolics (that exhibited greater toxicity against the non-resistant cells), suggesting the existence of synergistic or additive effects between the extracts’ components [89] (Table 3). 

Moreover, methanolic extracts of *Thymus schimperi*, containing high amounts of the flavone luteolin and derivatives, required doses of 50–100 μg/mL to inhibit the growth of human gastric adenocarcinoma AGS cells by 50% (IC_50_ = 88 μg/mL) [91]. In addition, Galasso et al. [42] reported that *T. longicaulis* extracts, containing high amounts of quercetin-*O*-hexoside, luteolin-*O*-hexoside and salvianolic acid K, caused a decrease of cell viability on breast cancer MDA-MB-23, colon carcinoma HCT-116 and lung fibroblast MRC-5 cells, equal to or even higher than those observed for vinblastine, a known anti-cancer agent [25]. Besides, Esmaeili-Mahani et al. [30] reported that polar extracts of *Thymus caramanicus* (at 150–200 μg/mL), could reduce the viability of human breast adenocarcinoma MCF-7 for about 60% and interestingly, its concomitant treatment (40 and 80 μg/mL extract) with the vincristine effectively potentiated the effect of the anticancer drug. Aqueous extracts of *T. satureioides*, mainly composed of rosmarinic acid and of luteolin glucoside, exhibited an IC_50_ value of 37.5 µg/mL in the same cell line [43]. The cytotoxicity capacity was also demonstrated for methanolic extracts of *T. serpyllum*, which caused a viability decrement of two human breast cancer cell lines (MCF-7 and MDAMB-231) after 72 h exposure (IC_50_ of 509 and 276 μg/mL, respectively), disclosing less anti-proliferation ability in human normal breast cells (MCF-10A), which further proved the anticancer property of this extract [92]. Polar extracts obtained from *T. carnosus Boiss.* were also tested for their cytotoxicity in MCF-7, BT-474 and RAW 264.7 cells using Alamar Blues assay, with best results being obtained for the hydroethanolic extracts rich in salvianolic acids and rosmarinic acid [69]. *T. zygis* extracts, containing similar phenolic composition, displayed promising results in Caco-2 and HepG2 cells (IC_50_ = 85.01 and 82.19 µg/mL, respectively), especially at 48 h of exposure [60]. The same group reported high cytotoxic ability of aqueous decoction and hydroethanolic extracts from different *Thymus* species towards Caco-2, namely *T. pulegioides* (IC_50_ of 82.25 and 105.44 µg/mL respectively) [61] and *T. citriodorus* (IC_50_ of 159.40 and 114.6 µg/mL respectively) [51], albeit both were less active against HepG2 cells [51]. In addition, aqueous decoction/hydroethanolic extracts of *T. mastichina* (rich in salvianolic acid derivatives) required low doses to inhibit the same cells growth by 50% (IC_50_ of 95.65 and 51.30 μg/mL towards Caco-2; 285.03 and 180.10 μg/mL towards HepG2 cells, respectively) [54]. Interestingly, *T. vulgaris*, the most studied and widely used thyme species worldwide, showed the lowest cytotoxic activity against Caco-2 and HepG2 cells after 48 h, for aqueous decoction (IC_50_ values of 376.8 and > 500 µg/mL, respectively) and hydroethanolic extracts (IC_50_ values of 442.45 and 254.25 µg/mL, respectively) [51].

In addition, Gordo et al. [88] reported that the dichloromethane and ethanol extracts of the aerial parts of *T. mastichina* were able to inhibit the viability of the HCT colon cancer cell line (IC_50_ value of 2.8 μg/mL and 12.0 μg/mL, respectively). Moreover, in this study, the authors also described that ursolic acid and a mixture (1:1) of oleanolic acid and ursolic acid, isolated in the chromatographic procedures, exhibited high cytotoxicity (IC_50_ of 6.8 and 2.8 μg/mL, respectively), suggesting that phenolic compounds maybe related to the protective effect of this thyme extract against colon cancer cells [88]. Discrepant results were reported recently for an ethanolic extract of *T. algeriensis Boiss. and Reut.* since this was ineffective in suppressing cancer cell growth (required a concentration greater than 10 000 μg/mL for achieving 50% reduction of cancer cell lines survival) [45]. In turn, other study using a methanolic extract from the same species, rich in gallic and vanillic acids, reported an effective protection against in vitro Multiple Myeloma cell line (U266) proliferation in a dose-dependent manner [90].

The DNA repair ability is very important, as DNA damage can lead to carcinogenesis if replication proceeds without a proper repair. The DNA-protective effect of plant extracts against DNA strand breaks induced by the oxidant agents H_2_O_2_ and 2,3-dimethoxy-1,4-naphthoquinone (DMNQ) has been performed by the comet assay, based in single cell gel electrophoresis [59,93]. Kozics et al. reported the protective effects of ethanolic extract of *T. vulgaris* (rich in rosmarinic acid) against DNA damage induced by strong oxidants, achieved the highest DNA-protective capacity at concentrations of 0.5 and 0.1 mg/mL, respectively [59]. 

Important effects of *Thymus* extracts in key mechanisms of tumor formation/progression (e.g., apoptosis and angiogenesis) have also been attested, both in in vitro and in vivo models. Although less studied than essential oils [8], phenolic-rich extracts from *Thymus* have been evaluated for effects on apoptosis of proliferative cells by cycle analysis, mostly through the flow cytometry method [94]. Some studies also investigated the apoptosis induction of cancer cells through activation of the caspase cascade or by disruption in the balance between pro- and antiapoptotic Bcl-2 family members, after exposure to thyme polar extracts. For example, Al-Menhali et al. [95] registered moderate apoptotic capacity of aqueous extracts of *T. vulgaris*, which were shown to increase the activity of caspase 3/7 on human colon carcinoma cells HCT116 in a concentration-dependent manner (3.5%, 36.1% and 54.7% increase in the activity of caspase 3/7 in cells treated with 0.2, 0.4, or 0.6 mg/mL, respectively) [95]. In addition, Esmaeili-Mahani et al. [30] highlighted the anticancer activity of *T. caramanicus* hydroalcoholic extracts, based on their ability to induce apoptosis in human breast cancer cells MCF-7 through the increment of caspase-3 and Bax and decreasing of Bcl-2 expression. These *T. caramanicus* extracts (200–250 µg/mL) also potentiated the cytotoxicity of vincristine, which was accompanied by an increase in the levels of cleaved caspase-3 [30]. Moreover, treatment of MDA-MB-231 breast cancer cells for 72 h with a methanolic extract of *T. serpyllum* gradually increased the caspase 3/7 enzyme activity (increased 1.6-, 2.2-, and 3-fold at 10, 250, and 500 μg/mL of extract, respectively) [92]. Less promising results were reported by Caprioli and coworkers [94] for the apoptotic effect of an ethanolic extract of *Thymus lanceolatus* (rich in rosmarinic acid), which required a concentration of 500 μg/mL for a significant increase in programmed cell death of chronic myelogenous leukemia K562 cells (11.5% and 14.2% at 48 and 72 h, respectively), with no significant effect towards colorectal adenocarcinoma CaCo-2 and neuroblastoma SH-SY5Y cells.

Once the inhibition of angiogenesis has been recognized to be advantageous for prevention of inflammation and neoplastic growth [33,96], methanolic extracts of *T. capitatus* (100 μg/mL) were shown to inhibit significantly micro-vessel formation in rat aortic rings [97]. Still, as the authors did not find significant effects against proliferation and migration of the human endothelial EA.hy926 cells after extract exposure, they concluded that the in vivo observed anti-angiogenic effects must occur thorough an indirect mechanisms of action [97].

The activity of enzymes involved in inflammation, such as COX-2 and its product PGE-2 (known as carcinogenesis promoters), and others involved in enhancing detoxification, like as GPx, SOD or catalase [34,35], and cytokines or others immunological mediators, have also been used for evaluation of the antitumoral activity of *Thymus* extracts [42,59]. With this regard, ethanolic extracts of *T. vulgaris* (1 and 0.5 mg/mL), with high amounts of rosmarinic acid, were described to induce a significant increase of GPx activity in human HepG2 cells, while SOD activity was significantly lowered with respect to the control [59]. Likewise, hydroalcoholic extracts of *T. longicaulis*, particularly rich in rosmarinic acid and methylapigenin, exhibited a strong anti-inflammatory and anticancer effectiveness through inhibition of COX-2 gene expression on human leukemia cell line THP-1 macrophages [42]. These results suggest that the downregulation of COX-2 expression/activity and growth inhibition of tumor cells are closely associated, determining the relationships of anti-inflammatory, antioxidant and cytotoxic potential [42,98].

Moreover, a water extract of *Thymus pannonicus* was demonstrated to cause a significant increase in the activity of xanthine oxidase, glutathione reductase and GPx in Ehrlich ascites carcinoma mice cells (EACs), having the authors concluded that the induction of oxidative stress may play an important role in antitumor properties of the extract [99]. The authors also demonstrated that this *Thymus* extract caused a strong tumor growth inhibition in mice, which was followed by a high percentage of apoptotic/necrotic cells in a dose dependent manner, as well as significant decrease (96–98%) in the EAC volume [99]. The extract was particularly rich in rosmarinic acid, salvianolic acid H, catechin derivatives and glycosidic derivatives of luteolin and apigenin. On another study, Caproli and coworkers also reported that an ethanolic extract of *T. lanceolatus*, rich in rosmarinic acid and flavonoids, was an efficient ROS scavenger, exhibiting a good antioxidant activity (since it decreases the ROS generation by about 30% in SH-SY5Y (neuroblastoma) and 70% in CaCo-2 (colorectal adenocarcinoma) and K562 (chronic myelogenous leukemia) cells [94].

## 5. Conclusions

The *Thymus* genus is a rich source of secondary metabolites with claimed health benefits. For the last years, phenolic-rich extracts from distinct *Thymus* species have been the focus of attention and screened for distinct bioactive properties of interest for application in industrial fields like food, pharmaceutical and cosmetics. This review highlights the biological activities of phenolic-rich extracts from various *Thymus* species, with emphasis devoted to antioxidant, anti-inflammatory and anticancer properties. Globally, the documented data revealed that *Thymus* extracts, rich in phenolic acids and flavonoids compounds, may have enormous ability to scavenge radicals and reduce inflammatory events. Importantly, several studies disclose that thyme extracts have the capacity to inhibit diverse proinflammatory mediators, such as cytokines (IL-1β, IL-6, IL-12, INF-γ, TNF-α), enzymes (iNOS, COX-2) and others, which are majorly regulated by the transcriptional factor NF-κB, whose activation is also associated with increased ROS generation. Moreover, there is also a considerable amount of work reflecting the promising cytotoxic activity of some *Thymus* phenolic-riche extracts on tumor cell lines, as well as their ability as regulators of the tumor cell cycle, leading to programed cell death or inhibiting angiogenesis. Hence, the evidenced health-benefits of *Thymus* extracts as antioxidant and anti-inflammatory agents corroborate their potential applications for pharmaceutical purposes, including the development of antitumoral compounds.

Regardless that, one must remark that so far, most of the gathered investigation has been based on in vitro experiments whereas there is a huge lack of in vivo studies, in order to be able to confirm the beneficial effects of *Thymus* polar extracts on human health.

## Figures and Tables

**Table 1:** Antioxidant properties of phenolic-rich extracts of distinct *Thymus* species.

*Thymus* Plants	Origin	Solvent Extraction (Major Components or TPC)	Results of Screen Assay	Ref
*T. algeriensis*	Algeria	EtOH (DHA, Rut, Epi)	DPPH (EC_50_, mg/mL) = 1.56 (EtOH), 1.68 (BHA), 0.002 (AA)/ABTS (EC_50_, mg/mL) = 1.74 (EtOH), 0.003 (Tlx), 0.001 (AA)	[45]
	Algeria	MeOH-H_2_O (RA, CaffeoylRA, Kaemp, Eri-Glc)	Plasma antioxidant capacity (DPPH, 800 mg/kg bw): 22% of Inhib (treated group)/6 % (non-treated group) Ferric reducing ability of plasma (FRAP, 800 mg/kg bw): 908 µM FeSO_4_ eq/mL (treated group)/405 µM FeSO_4_/mL (non-treated group) ↑ CAT activity; ↑ GSH levels (400 and 800 mg/kg bw); ↓ MDA levels (200 and 400 mg/kg bw)	[46]
	Tunisia	MeOH (Carvacrol, RA, Tetramethyl-scutellarein, Kaemp-*O*-Hexu)	DPPH (EC_50_, μg/mL): 8.9–68.8/β-carot bleach (mg/mL): 0.03–1.81/FRAP (mmol Fe^2+^/L): 0.3–20.6	[47,48]
*T. atlanticus*	Morocco	H_2_O (RA, CaffA, Quer)	DPPH (EC_50_, μg/mL) = 120 (H_2_O), 510 (Tlx)/FRAP (mmol Tlx/g extract) = 40.0 (H_2_O), 44.3 (Tlx)	[43]
*T. caespititius*	Portugal	H_2_O (RA, Lut-*O*-Glr)	DPPH (EC_50_, μg/mL) = 13.8 (H_2_O), 6.90 (AA)/RP (EC_50_, μg/mL) = 39.3 (H_2_O), 16.30 (BHA)/β-carot bleach (EC_50_ μg/mL) = 6.1 (H_2_O), 0.4 (BHA)	[40]
*T. camphoratus*	Portugal	DE, EA, *n*-but, H_2_O [TPC (GAE mg/mL) = 10.77 (DE), 10.21 (EA), 6.62 (*n*-but), 1.82 (H_2_O)]	DPPH (EC_50_ μg/mL): 3.1 (DE), 2.7 (EA), 6.4 (*n*-but), 3.2 (H_2_O)/O_2_- scav (EC_50_ μg/mL): 7.8 (DE), 11.0 (*n*-but), 9.5 (H_2_O)	[49]
*T. capitatus*	Greece	70% MeOH (RA, FA, Nar, Lut); H_2_O (RA, CaffA, Epi, Epig)	DPPH (mg TE/g DW): 56.2/ABTS (mg TE/g DW): 75.2/FRAP (mg TE/g DW): 76.1	[50]
*T. carnosus*	Portugal	DE, EA, *n*-but, H_2_O [TPC (GAE mg/mL) = 3.55 (DE), 5.97 (EA), 2.99 (*n*-but), 1.24 (H_2_O)]	DPPH (EC_50_ μg/mL): 4.0 (DE), 3.0 (EA), 5.2 (*n*-but), 3.6 (H_2_O)/O_2_- scav (EC_50_ μg/mL): 12.3 (DE), 8.9 (EA), 8.9 (*n*-but), 13.6 (H_2_O)	[49]
*T. citriodorus*	Portugal	EtOH-H_2_O (RA, SA I, Lut-*O*-Hexu) H_2_O (RA, Lut-*O*-Hexu)	ABTS (EC_50_ mmol Tlx eq./g extract): 1.52 (EtOH-H_2_O); 1.21 (H_2_O)/OH scav (% inhib, 1 mg/mL): 37.97 (H_2_O)	[51]
		EtOH-H_2_O (RA, Lut-Glr Api-Glr)	Intracellular ROS formation on HepG2 cells (EtOH-H_2_O 50 μg/mL): ↓ 21% (at 5 μM potassium dichromate-stimulated cells); ↓ 20% (at 25 μM potassium dichromate-stimulated cells)	[52]
*T. fragrantissimus*	Portugal	H_2_O (RA, Lut-*O*-Glr, CaffeoylRA)	DPPH (EC_50_, μg/mL) = 12.9 (H_2_O), 6.90 (AA)/RP (EC_50_, μg/mL) = 33.4 (H_2_O), 16.30 (BHA)	[41]
*T. herba-barona*	Portugal	H_2_O (RA, Lut-*O*-Glr, CaffeoylRA, SA B)	DPPH (EC_50_, μg/mL) = 11.6 (H_2_O), 6.90 (AA)/RP (EC_50_, μg/mL) = 35.1 (H_2_O), 16.30 (BHA)/β-carot bleach (EC_50_ μg/mL) = > 26.7 (H_2_O), 0.4 (BHA)	[40]
*T. longicaulis*	Croatia	EtOH (PC: THA = 5.41%; TFlav = 0.40%)	DPPH (EC_50_ μg/mL): 3.01 (EtOH), 0.66 (RA),0.73 (Lut), 1.67 (Tlx)/RP (EC_50_ μg/mL): 11.8 (EtOH), 2.67 (RA), 4.51 (Lut), 6.64 (Tlx)/TBARS (EC_50_ μg/mL): 34.3 (EtOH), 21.1 (RA), 2.03 (Lut)	[4]
	Italy	EtOH-H_2_O (RA, SA K, Lut-*O*-Hex, Quer-*O*-Hex)	DPPH (EC_50_, μg/mL): 9.5 (H_2_O-MeOH), 5.1 (Tlx)/ABTS (EC_50_, μg/mL): 9.5 (H_2_O-MeOH), 5.1 (Tlx)/RP (μM TE/g extract): 475 (H_2_O-MeOH)/ORAC (μM TE/g extract): 776.5 (H_2_O-MeOH)	[42]
*T. mastichina*	Portugal	DE, EA, *n*-but, H_2_O [TPC (GAE mg/mL) = 26.28 (DE), 19.50 (EA), 9.74 (*n*-but), 2.23 (H_2_O)]	DPPH (EC_50_ μg/mL): 2.7 (DE), 3.7 (EA), 4.0 (*n*-but), 3.9 (H_2_O)/O_2_- scav (EC_50_ μg/mL): 10.0 (DE), 4.9 (EA), 6.9 (*n*-but), 12.2 (H_2_O)	[49]
		MeOH, H_2_O [TPC (mg GAE/g) = 165.29; TF (mg CE/g) = 83.85]	DPPH (EC_50_ mg/mL): 0.69 (MeOH), 2.57 (H_2_O), 0.04 (Tlx)/RP (EC_50_ mg/mL): 0.23 (MeOH), 0.7 (H_2_O), 0.03 (Tlx)/β-carot bleach (EC_50_ mg/mL): 0.9 (MeOH), 0.003 (Tlx)/TBARS (EC_50_ mg/mL): 0.43 (MeOH), 0.004 (Tlx)	[53]
	Portugal	EtOH-H_2_O (RA, SA I) H_2_O (RA, SA I)	ABTS (EC_50_ mmol Tlx eq./g extract): 1.48 (EtOH-H_2_O); 0.96 (H_2_O)/OH scav (% inhib, 1 mg/mL): 43.22 (EtOH-H_2_O); 48.52 (H_2_O)	[54]
	Spain	50% MeOH (RA, CaffA, Lut, Lut-Glc)	DPPH (mg TE/g DW): 18–149/FRAP (mg TE/g dw): 30–154, different populations	[37]
*T. nummularius*	Turkey	MeOH (QA, RA, Lut, Kaemp)	DPPH (EC_50_ μg/mL): 5.73 (MeOH), 1.21 (RA), 47.1 (BHT), 19.6 (α-Toc)/ABTS (EC_50_ μg/mL): 7.1 (MeOH), 1.7 (RA), 10.9 (BHT)/β-carot bleach (EC_50_ μg/mL): 6.54 (MeOH), 12.1 (RA), 9.95 (BHT)	[44]
*T. praecox subsp. polytrichus, T. serpyllum subsp. serpyllum, T. striatus*	Croatia	EtOH *T. praecox* [PC: THA = 54.39%; TFlav = 0.24%)/EtOH *T. serpyllum* (PC: THA = 4.36%; TFlav = 0.4%)/EtOH *T. striatus* (PC: THA = 3.35%; TFlav = 0.15%)	DPPH (EC_50_ μg/mL): 3.4 (*T. praecox*), 4.06 (*T. striatus*), 6.01 (*T. serpyllum*) 0.73 (Lut), 1.67 (Tlx)/RP (EC_50_ μg/mL): 15.1 (*T. praecox*), 14.7 (*T. striatus*), 14.5 (*T. serpyllum*), 2.67 (RA), 4.51 (Lut), 6.64 (Tlx)/TBARS (EC_50_ μg/mL): 78.7 (*T. praecox*), 63.0 (*T. striatus*), 80.0 (*T. serpyllum*), 21.1 (RA), 2.03 (Lut)	[4]
*T. pseudolanuginosus*	Portugal	H_2_O (RA, Lut-*O*-Glr, SA B)	DPPH (EC_50_, μg/mL) = 10.9 (H_2_O), 6.90 (AA)/RP (EC_50_, μg/mL) = 32.2 (H_2_O), 16.30 (BHA)/β-carot bleach (EC_50_ μg/mL) = 2.4 (H_2_O), 0.4 (BHA)	[40]
*T. pulegioides*	Croatia	EtOH (PC: THA = 6.17%; TFlav = 0.42%)	DPPH (EC_50_ μg/mL): 4.18 (EtOH), 0.66 (RA), 0.73 (Lut), 1.67 (Tlx)/RP (EC_50_ μg/mL): 11.4 (EtOH), 2.67 (RA), 4.51 (Lut), 6.64 (Tlx)/TBARS (EC_50_ μg/mL): 34.8 (EtOH), 21.1 (RA), 2.03 (Lut)	[4]
	Portugal	MeOH [TPC (mg GAE/g) = 210.49; TFlav (mg CE/g) = 128.24; TFlol (mg QE/g) = 126.74)]	DPPH (EC_50_ μg/mL): 680/RP (EC_50_ μg/mL): 490/β-carot bleach (EC_50_ μg/mL): 30/TBARS (EC_50_ μg/mL): 220	[55]
	Portugal	H_2_O (RA, Lut-*O*-Glr, CaffeoylRA)	DPPH (EC_50_, μg/mL) = 9.5 (H_2_O), 6.90 (AA)/RP (EC_50_, μg/mL) = 30.7 (H_2_O), 16.30 (BHA)	[41]
*T. satureioides*	Morocco	H_2_O (RA, CaffA, Quer)	DPPH (EC_50_, μg/mL) = 440 (H_2_O), 510 (Tlx)/FRAP (mmol Tlx/g extract) = 40.1 (H_2_O), 44.3 (Tlx)	[43]
*T. sipyleus* Boiss. subsp. *rosulans*	Morocco	H_2_O [TPC (mg GAE/g) = 147.6 (decoction), 118.5 (infusion)]	DPPH (EC_50_, μg/mL) = 43.5 (decoction), 87.38 (infusion), 27.63 (AA)	[56]
*T. vulgaris*	Algeria	MeOH-H_2_O (TPC (mg GAE/100g DW) = 81.5; RA, Flav)	DPPH (EC_50_ μg/mL): 1.78/ABTS (EC_50_ μg/mL): 0.69/OH scav (EC_50_ μg/mL): 0.24	[57]
	Croatia	EtOH (PC: THA = 3.58%; TFlav = 0.24%)	DPPH (EC_50_ μg/mL): 5.6 (EtOH), 0.66 (RA),0.73 (Lut), 1.67 (Tlx)/RP (EC_50_ μg/mL): 14.1 (EtOH), 2.67 (RA), 4.51 (Lut), 6.64 (Tlx)/TBARS (EC_50_ μg/mL): 69.6 (EtOH), 21.1 (RA), 2.03 (Lut)	[4]
	Egypt	EtOH [TPC (mg/g) = 212; TFlav (mg/g) = 85)	Protective antioxidant effects against lead intoxicated rats: ↓ GSH, GPx and CAT levels; ↑ MDA levels	[58]
	Portugal	EtOH-H_2_O (RA, SA I, Lut-*O*-Hexu) H_2_O (RA, Lut-*O*-Hexu)	ABTS (EC_50_ mmol Tlx eq./g extract): 0.92 (EtOH-H_2_O); 0.79 (H_2_O)/OH scav (% inhib, 1 mg/mL): 9.58 (H_2_O)	[51]
	Slovakia	H_2_O, MeOH (RA, SA K isomer, Lut-Hex, Eri-Glc)	DPPH (EC_50_, μg/mL): 44.7 (MeOH), 2.79 (Que)/ABTS (EC_50_ μg/mL): 13.8 (MeOH), 49.6 (H_2_O), 1.17 (Que)	[59]
*T. zygis*	Morocco	H_2_O (RA, CaffA, Quer)	DPPH (EC_50_, μg/mL): 440 (H_2_O), 510 (Tlx)/FRAP (mmol Tlx/g extract): 65.0 (H_2_O), 44.3 (Tlx)	[43]
	Portugal	H_2_O (RA, CaffeoylRA)]	DPPH (EC_50_, μg/mL) = 12.6 (H_2_O), 6.90 (AA)/RP (EC_50_, μg/mL) = 33.7 (H_2_O), 16.30 (BHA)	[41]
		EtOH-H_2_O (RA, SA I, SA K) H_2_O (RA, Lut-*O*-Hex, Lut-*O*-Hexu)	ABTS (EC_50_ mmol Tlx eq./g extract): 1.08 (EtOH-H_2_O); 0.76 (H_2_O)/OH scav (% inhib, 1 mg/mL): 66.28	[60]

AA—ascorbic acid; ABTS—2,2′-azino-bis-(3-ethylbenzothiazoline-6-sulphonic acid) radical scavenging assay; BHA—2(3)-t-butyl-4-hydroxyanisole; BHT—3.5-di-tert-butyl-4 hydroxytoluene; CaffA/CaffA deriv—caffeic acid/caffeic acid derivative; CE—chatequin equivalents; β-carot bleach—β-carotene/linoleic acid bleaching inhibition assay; CAT—catalase; DE—diethyl ether; DHA—dihydroxybenzoic acid; DPPH—1,1-diphenyl-2-picrylhydrazyl radical scavenging assay; DW—dry weight; EA—ethyl acetate; Epi—epicatechin; Epig—epigallocatechine; Eri—eriodictyol; EtOH—ethanol; FA- ferulic acid; Flav—flavonoids; FRAP—ferric reducing antioxidant power; GAE—gallic acid equivalent; Glc—glucoside; Glr—glucuronide; GPx—glutathione peroxidase; GSH—reduced glutathione; Hex—hexoside; Hexu—hexuronide; Kaemp—kaempferol; Lut—luteolin; MDA—malonaldehyde; MeOH—methanol; Nar—naringin; *n*-but—*n*-butanol; ND—not determined; OH scav—hydroxyl radical scavenging assay; O_2_- scav—O_2_- radical scavenging assay; ORAC—oxygen radical absorbance capacity; QA—quinic acid; Quer—quercetin; QE—quercetin equivalents; RA—rosmarinic acid; RP—reducing power; Rut—rutin; SA—salvianolic acid; TBARS—thiobarbituric acid reactive substances assay; TE—trolox equivalente; TFlav—total flavonoids; TFlol—total flavanols; THA—total hydroxycinnamic acids; Tlx—trolox; TPC—total phenolic compounds; PC—phenolic compounds; α-Toc—α-tocopherol.

**Table 2:** Anti-inflammatory properties of phenolic-rich extracts of distinct *Thymus* species.

Plant Species	Origin	Solvent Extraction (Major Components or TPC)	Screen Assay	Effect	Ref
*T. algeriensis*	Algeria	MeOH-H_2_O (SA K, RA-Glc, Lut-Glr)	COX Inhib 5-LOX Inhib Carrageenan-Induced Hind-Paw Edema Model Carrageenan-Induced Leukocyte Migration (LeucM)	EC_50_ (μM) = COX-1: 12.4 (MeOH-H_2_O), 4.06 (dic); COX-2: 0.05 (MeOH-H_2_O), 0.06 (cel); 5-LOX: 2.7 (MeOH-H_2_O), 3.2 (zil) Paw Edema: MeOH-H_2_O (200 mg/kg) = ↓~15% LeucM: MeOH-H_2_O (600 mg/kg) = ↓62%; dic (20 mg/kg) = ↓39%; dexa (2 mg/kg) = ↓30%	[68]
*T. atlanticus*	Morocco	H_2_O (RA, CaffA, Quer)	Croton oil-induced mice ear edema/Carrageenan-induced rat paw edema	At 900 µg/ear after 8 h: ↓ 84.6% (in comparison to ind)/At 50 mg/kg after 5 h: ↓ 9.5% (in comparison to ind)	[43]
		H_2_O (ND)	Inhib of denaturation of bovine serum albumin/Inhib of erythrocyte lysis	EC_50_ μg/mL: 122.9 (H_2_O), 86.07 (ind)/EC_50_ μg/mL: 93.28 (H_2_O), 97.83 (ind)	[62]
*T. caespititius*	Portugal	H_2_O (RA, Lut-*O*-Glr)	5-LOX inhib/NO scav	5-LOX (EC_50_ μg/mL): 590.5 (H_2_O), 7.8 (AA)/NO^●^ (EC_50_ μg/mL): 229.7 (H_2_O), 228.0 (AA)	[40]
*T. camphoratus*, *T. carnosus*	Portugal	DE, EA, *n*-but [TPC (GAE mg/mL) = 10.77 (DE), 10.21 (EA), 6.62 (*n*-but)]	5-LOX inhib	5-LOX (EC_50_ μg/mL): 29.9 (DE), 27.4 (EA), 28.0 (*n*-but) (*T. camphoratus*); 23.5 (DE), 29.6 (EA), 18.3 (*n*-but) (*T. carnosus*)	[49]
*T. carnosus Boiss.*	Portugal	H_2_O (SA A isomer, SA K) EtOH-H_2_O (SA A isomer, SA K, RA)	NO scav in LPS-induced macrophages	H_2_O (200 μg/mL) = ↓ 90% of control; EtOH-H_2_O (15 μg/mL) = ↓ 75% of control	[69]
*T. fontanesii*	Algeria	MeOH-H_2_O (Carnosol, Salvigenin)	COX Inhib, 5-LOX Inhib/Carrageenan-Induced Hind-Paw Edema Model/Carrageenan-Induced Leukocyte Migration (LeucM)	EC_50_ (μM) = COX-1: 12.88 (MeOH-H_2_O), 4.06 (dic); COX-2: 0.04 (MeOH-H_2_O), 0.06 (cele); 5-LOX: 2.5 (MeOH-H_2_O), 3.2 (zil)/Paw Edema: MeOH-H_2_O (600 mg/kg) and dic (20 mg/kg) = ↓ 44%/LeucM: MeOH-H_2_O (600 mg/kg) = ↓ 52%; dic (20 mg/kg) = ↓ 39%; dexa (2 mg/kg) = ↓ 30%	[68]
*T. herba-barona*	Portugal	H_2_O (RA, Lut-*O*-Glr, CaffeoylRA, SA B)	5-LOX inhib/NO scav	5-LOX (EC_50_ μg/mL): 840.8 (H_2_O), 7.8 (AA)/NO (EC_50_ μg/mL): 286.1 (H_2_O), 228.0 (AA)	[40]
*T. longicaulis*	Italy	EtOH-H_2_O (RA, SA K, Lut-*O*-Hex, Quer-*O*-Hex)	COX-2 gene expression on THP-1 cells	At 50 µg/mL: ↓ 42% (extract collected in october)	[42]
*T. mastichina*	Portugal	DE, EA, *n*-but [TPC (GAE mg/mL) = 10.77 (DE), 10.21 (EA), 6.62 (*n*-but)]	5-LOX inhib	5-LOX (EC_50_ μg/mL): 62.5 (DE), 53.1 (EA), 30.5 (*n*-but)	[49]
*T. praecox* subsp. *skorpilii* var. *skorpilii*	Turkey	MeOH fraction (CA, Lut-*O*-Glc, 3-*O*-feruloylQA, Quer-*O*-Hex)	Carrageenan-induced paw edema	% inhib (MeOH f, 100 mg/kg) = 4.26% (1 h), 67.67% (2 h), 52.07% (3 h), 65.75% (4 h); % inhib (ind 5, mg/kg) = 34.04% (1 h), 88.65% (2 h), 82.89% (3 h), 88.63% (4 h)	[70]
*T. pseudolanuginosus*	Portugal	H_2_O (RA, Lut-*O*-Glr, SA B)	5-LOX inhib/NO scav	5-LOX (EC_50_ μg/mL): 813.6 (H_2_O), 7.8 (AA)/NO (EC_50_ μg/mL): 298.98 (H_2_O), 228.0 (AA)	[40]
*T. satureioides*	Morocco	H_2_O (ND)	Inhib of denaturation of bovine serum albumin/Inhib of erythrocyte lysis	EC_50_ μg/mL: 181.42 (H_2_O), 86.07 (ind)/ EC_50_ μg/mL: 204.41 (H_2_O), 97.83 (ind)	[62]
		H_2_O (RA, Lut-7-Glc, Hesp)	Croton oil-induced mice ear edema	At 900 µg/ear after 8 h: ↓ 29.7% (in comparison to ind)	[43]
*T. sipyleus* Boiss. subsp. *rosulans*	Morocco	H_2_O [TPC (mg GAE/g) = 147.6 (decoction), 118.5 (infusion)]	Inhib of NO and TNF-α production in LPS-induced macrophages	At 50 μg/mL: ↓ NO: 50.86% (decoction) and 47.79% (infusion); ↓ TNFα: 49.76% (decoction) and 54.79% (infusion)	[56]
*T. vulgaris*	United Kingdom	H_2_O [TPC (mg GAE/g herb) = ~20]	IL-8 release of PBLs prior to stimulation by TNFα and H_2_O_2_	↓IL-8 release in 35 and 37% upon stimulation of TNF-α and H_2_O_2_, respectively	[71]
	Brazil	PG (Thymol, Carvacrol, Linalool, Geranoil, Citral, Tannins, Organic acids, Flavonoids)	Cytokines production by LPS-induced RAW264.7 macrophages	IL-1β (pg/mL) = 28, 2, 2 at 25, 50 and 100 mg/mL extract, respectively; TNF-α (pg/mL) = 4466, 824, 12 at 25, 50 and 100 mg/mL extract, respectively	[72]
	Egypt	EtOH [TPC (mg/g) = 212; TFlav (mg/g) = 85)]	Lead intoxicated rats	Protective effects against lead intoxicated rats: ↑ IL-1β, IL-6 and TNF-α levels; ↓ IL-10 and (IFN)-γ levels	[58]
*T. zygis*	Morocco	H_2_O (RA, CaffA, Lut-7-*O*-Glc)	Croton oil-induced mice ear edema/Carrageenan-induced rat paw edema	At 900 µg/ear after 8 h: ↓ 70% (in comparison to ind)/At 50 mg/kg after 5 h: ↓ 3.7% (in comparison to ind 10 mg/kg)	[43]
		H_2_O (ND)	Inhib of denaturation of bovine serum albumin/Inhib of erythrocyte lysis	EC_50_ μg/mL: 133.25 (H_2_O), 86.07 (ind)/EC_50_ μg/mL: 156.20 (H_2_O), 97.83 (ind)	[62]
	Portugal	EtOH-H_2_O (RA, SA I, SA K); H_2_O (RA, Lut-*O*-Glc, L-*O*-Hexu)	NO scav/Inhib of NO production in LPS-induced macrophages	NO scav (% inhib): 29.32 (H_2_O)/At 50 µg/mL: ~89% (EtOH), 48% (H_2_O-H_2_O)	[60]

CA—chlorogenic acid; CaffA—caffeic acid; Celecoxibe—cele; COX-2—cyclooxygenase; DE—diethyl ether; Dex—dexamethasone; Dic—diclofenac; EA—ethyl acetate; EtOH—ethanol; GAE—allic acid equivalent; Glc—glucoside; Glr—glucuronide; Gly—glucosyl; Hesp—hesperetin; Hex—hexoside; Hexu—hexuronide; IFN—interferon γ; IL—interleukin; LOX—lipoxygenase; Inhib—Inhibition; Ind—indomethacin; LPS—lipopolysaccharide; Lut—luteolin; MeOH—methanol; *n*-but—*n*-butanol; ND—not determined; NO—nitric oxide; PBL—peripheral blood lymphocytes; PG—propylene glycol; QA—quinic acid; Quer—quercetin; RA—rosmarinic acid; SA—salvianolic acid; Scav—scavenging; TFlav—total flavonoids; THP-1—human monocytic leukemia cell line; TNF-α—tumor necrosis factor-α; TFlav—total flavonoids; TPC—total phenolic compounds; Zil—zileuton.

**Table 3:** Anticancer properties of phenolic-enriched extracts of distinct *Thymus* species.

Plant Species	Origin	Solvent extraction (Major Components or TPC)	Screen Assay	Effect	Ref
*T. algeriensis*	NI	MeOH (GallicAc, VanillicAc)	CViab (MTT) on U266 cell line	CVI (%): ~15%	[90]
*T. caramanicus*	Iran	EtOH-H_2_O (Carvacrol, Thymol, Borneol, Cymene)	CViab (MTT) on MCF-7 cells/Biochemical markers of apoptosis and cell proliferation (Western blot)	CVI (%) = MCF-7: 85% (EtOH-H_2_O), 85% (Vin), 65% (EtOH-H_2_O+Vin) after 40 µg/mL extract; MCF-7: 70% (EtOH-H_2_O), 85% (Vin), 50% (EtOH-H_2_O+Vin), after 80 µg/mL extract/Western blot = MCF-7 after 200 µg/mL extract: ↑ caspase 3, ↑ bax, ↓Bcl2, ↓ cyclin D1	[30]
*T. carnosus Boiss.*	Portugal	H_2_O (SA A isomer, SA K) EtOH-H_2_O (SA A isomer, SA K, RA)	CViab (Alamar Blue) on MCF-7, BT-474, RAW 264.7	CVI (IC_50_ µg/mL, H_2_O) = MCF-7: 841.28 (24 h); 735.18 (48 h); BT-474: 533.87 (24 h); 603.86 (48 h); RAW 264.7: 603.07 (24 h); 223.22 (48 h); CVI (IC_50_ µg/mL, EtOH-H_2_O) = MCF-7: 86.87 (24 h); 74.37 (48 h); BT-474: 39.91 (24 h); 34.45 (48 h); RAW 264.7: 24.80 (24 h); 28.20 (48 h)	[69]
*T. citriodorus*	Portugal	EtOH-H_2_O (RA, SA I, Lut-*O*-Hexu) H_2_O (RA, Lut-*O*-Hexu)	CViab (Alamar Blue) on Caco-2 and HepG2	CVI (IC_50_ µg/mL, EtOH-H_2_O) = Caco-2: 128.2 (24 h); 114.6 (48 h); HepG2: > 500 (24 h); >500 (48 h); CVI (IC_50_ µg/mL, H_2_O) = Caco-2: 223.7 (24 h); 159.4 (48 h); HepG2: >500 (24 h); >500 (48 h)	[51]
*T. mastichina*	Portugal	EtOH-H_2_O; H_2_O (RA, SA A isomer, Lut-Hex, Quer-Hex)	CViab (Alamar Blue) on Caco-2 and HepG2 cells	CVI (IC_50_ µg/mL, EtOH-H_2_O) = Caco-2: 71.18 (24 h); 51.30 (48 h); HepG2: 264.60 (24 h); 180.10 (48 h); CVI (IC_50_ µg/mL, H_2_O) = Caco-2: 220.60 (24 h); 95.65 (48 h); HepG2: >500 (24 h); 285.03 (48 h)	[54]
*T. pulegioides*	Portugal	EtOH-H_2_O; H_2_O (RA, Lut-*O*-Hexu, Eri-*O*-Hexu, Chr-Hex)	CViab (Alamar Blue) on Caco-2 cells	CVI (IC_50_ µg/mL) = 82.25 (H_2_O); 105.44 (EtOH-H_2_O)	[61]
*T. satureioides*	Morocco	H_2_O (RA, Lut-*O*-Glc)	CViab (MTT) on MCF-7	CVI (IC_50_ µg/mL) = 37.5 ± 4.02	[43]
*T. schimperi*	Ethiopia	MeOH 70% (Lut, Lut-7-*O*-Glc, Lut-7-*O*-xy)	CViab (MTT) on AGS and HepG2 cells	CVI (IC_50_, µg/mL) = AGS: 88, after 50–100 µg/mL extract; HepG2: 326, after 200–400 µg/mL extract/CVI (%) = AGS: 38%, HepG2: 35%	[91]
*T. serpyllum* (*Ts*)*, T. vulgaris* (*Tv*)	Poland	*Ts* H_2_O (RA, CaffA, LAc, Lut-7-*O*-Glr, Lut-7-*O*-Rut, Eri-7-*O*-Rut) *Tv* H_2_O (RA, CaffA, Lut-7-*O*-Glr, Lut-7-*O*-Rut, Eri-7-*O*-Rut)	CViab (MTT) on MCF-7/Adr or wt cells	CVI (IC_50_, µg/mL) = MCF-7/Adr: 399 (*Ts*), 407 (*Tv*)/(IC_50_, mM) = MCF-7/Adr: 0.81 (RA), 1.26 (LAc), 1.81 (CaffA), 1.87 (Lut-7-*O*-Glr), 4.2 (Lut-7-*O*-Rut), 2.6 (Erd-7-*O*-Rut), 5.8 (Ab); MCF-7/wt: 0.74 (RA), 1.09 (Lut-7-*O*-Glr), 0.45 (LAc), 1.36 (CaffA), 18.2 (Lut-7-*O*-Rut), 1.71 (Eri-7-*O*-Rut), >1000 (Ab)CVI (%) = MCF-7/Adr: 60% (*Ts, Tv*), MCF-7/wt: 30% (*Ts*), 60% (*Tv*) at 500 mg/L extract/MCF-7/Adr: 86% (RA), 26% (Lut-7-*O*-Glr), MCF-7/wt: 92% (RA), 80% (LAc), 54% (Lut-7-*O*-Glr), at 1.25 mM	[89]
*T. serpyllum*	Turkey	MeOH (ND)	CViab (XTT) on MCF-7 and MDA-MB-231 cells/DNA fragmentation/caspase 3/7 enzyme activity	CVI (%) MCF-7: ↓ 15, 32, 42.5%, and 71 at 10, 100, 500, and 1000 μg/mL of extract, respectively CVI (%) MDA-MB-231: ↓ 22, 33, 66, and 75 at 10, 100, 500, and 1000 μg/mL of extract, respectively DNA fragmentation of MDA-MB-231 cells: ↑ 16%, 30%, and 55% at 10, 100, 250, and 500 μg/mL extract Caspase 3/7 enzyme activity: ↑ 1.6-, 2.2-, 3-fold at 10, 250, 500 μg/mL	[92]
*T. vulgaris*	Brazil	PG (Thymol, Carvacrol, Linalool, Geranoil, Citral, Tannins, Organic Acids, Flavonoids)	CViab (MTT, NR and CVA) on RAW 264.7, FMM-1, MCF-7 and HeLa cells	CVI, MTT (%) = RAW 264.7 and FMM-1: ↓ at 25, 50 and 100 mg/mL; MCF-7: ↓ at 50 and 100 mg/mL; HeLa cells: ↓ at 100 mg/mL extract CVI, NR (%) = RAW 264.7: ↓ at 50 and 100 mg/mL; FMM-1: ↓ no significant; MCF-7: ↓ at 25, 50 and 100 mg/mL; HeLa cells: ↓ at 50 and 100 mg/mL extract CVI, CVA (%): RAW 264.7: ↓ at 50 and 100 mg/mL; FMM-1: ↓ at 25, 50 and 100 mg/mL; MCF-7: ↓ at 100 mg/mL; HeLa cells: ↓ at 50 mg/mL extract	[72]
	Portugal	EtOH-H_2_O (RA, SA I, Lut-*O*-Hexu) H_2_O (RA, Lut-*O*-Hexu)	CViab (Alamar Blue) on Caco-2 and HepG2 cells	CVI (IC_50_ µg/mL, EtOH-H_2_O) = Caco-2: >500 (24 h); 442.45 (48 h); HepG2: 495.05 (24 h); 254.25 (48 h)/CVI (IC_50_ µg/mL, H_2_O) = Caco-2: >500 (24 h); 376.8 (48 h); HepG2: >500 (24 h); >500 (48 h)	[51]
	Slovakia	EtOH (RA, SA K isomer, Lut-Hex, Api-Glr)	CViab (MTT), DNA damage (comet assay); enzymatic activity of tumor HepG2 cells	CVI (IC_50_, mg/mL) = 4.3/HepG2 DNA damage induced by H_2_O_2_ and DMNQ: ↓ after 0.5 mg/mL extracts, at 24 h/Enzymatic activity: ↑ GPx, ↓ SOD, after 1 and 0.5 mg/mL extracts	[59]
	South Africa	Acet (ND)	CViab (XTT) on HeLa and non-tumor Vero cells	CVI (IC_50_, µg/mL) = HeLa: >200, 0.002 (AmD); Vero: 138, 0.027 (AmD)	[86]
*T. zygis* subsp. zygis	Portugal	EtOH-H_2_O (RA, SA I, SA K)	CViab (Alamar Blue) on Caco-2 and HepG2 cells	CVI (IC_50_ µg/mL) = Caco-2: 85.01 ± 15.10; HepG2: 82.19 ± 2.46 µg/mL	[60]

Ac—acid; Acet—acetone; Adr—adriamycin-resistant; AGS—human gastric adenocarcinoma cells; AmD—actinomycin D; Ab—arbutin; BT-474—breast cancer cell line; CaffA—caffeic acid; CaCo-2—colon adenocarcinoma cell line; Chr—chrysoeriol; CViab—cell viability; CVA—crystal violet assay; CVI—cell viability inhibition; DMNQ—2,3-dimethoxy-1,4-naphthoquinone; Eri—eriodictiol; FMM-1—human gingival fibroblasts; Glc—glucoside; Glr—glucuronide; GPx—glutathione peroxidase; Hex—hexoside; Hexu—hexuronide; HeLa—cervical carcinoma cells; HepG2—hepatocellular carcinoma cells; LAc—lithospermic acid; Lut—luteolin, MeOH—methanol; MCF-7—human breast carcinoma cells; MDA-MB-231—breast cancer cell line; MTT—3-(4,5-dimethylthiazol-2-yl)-2,5-diphenyltetrazolium bromide; ND—not determined; NI—not indicated; NR—neutral red assay; PG—propylene glycol; Quer—quercetin; RA—rosmarinic acid; Rut—rutinoside; SA—salvianolic acid; SOD—superoxide dismutase; U266—multiple myeloma cell line; Vin—vincristine; Wt—wild-type; Xy—xyloside; XTT—2,3-bis-(2-methoxy-4- nitro-5-sulfophenyl)-2H-tetrazolium-5-carboxanilide.
